# Supplementary material for: Composite Magnetic Filaments: From Fabrication to Magnetic Hyperthermia Application
Source: Micromachines (Basel). 2025 Mar 12;16(3):328. doi: 10.3390/mi16030328 (PMC11946111; doi:10.3390/mi16030328)
Supplement: Supplementary file 1 [file micromachines-16-00328-s001.zip › micromachines-3495248-supplementary.pdf]

Supplementary Materials

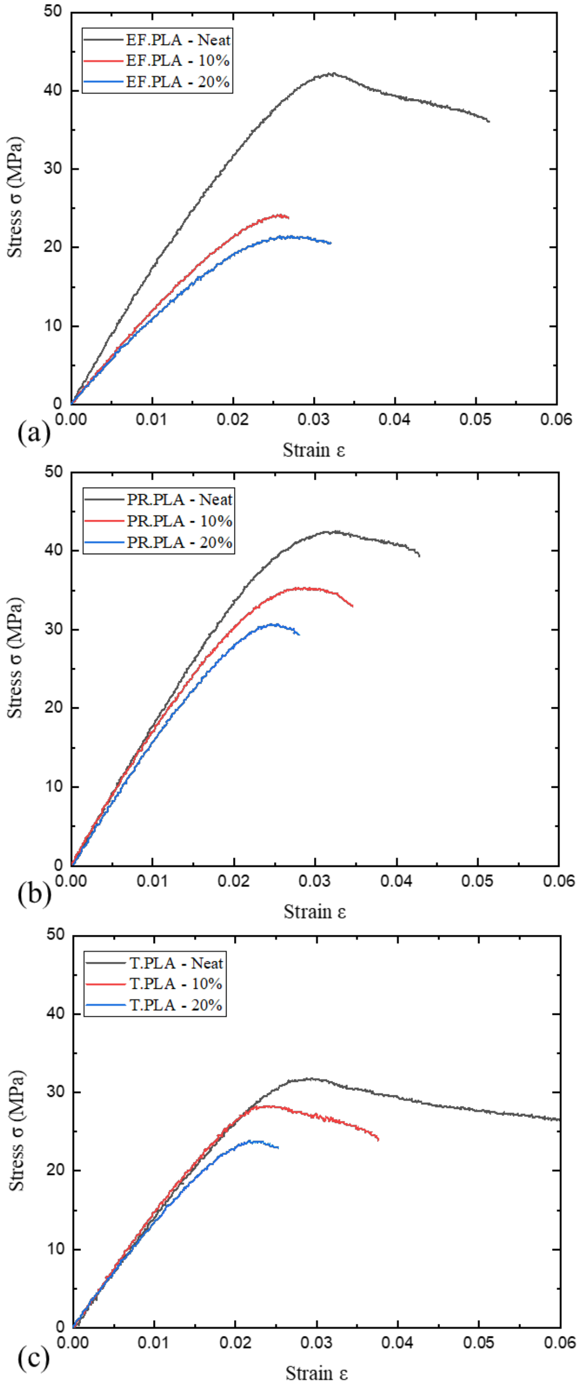

Figure S1: Stress-strain curves illustrating the mechanical behavior of (a) EF.PLA, (b) Pr.PLA, and (c) T.PLA materials as a function of increasing magnetite concentration.

Table S1. E is the elastic modulus, σ_{UTS} is the ultimate tensile strength, σy is the yield strength, ε is the elongation at break. ΔE is the percentage difference in elastic modulus compared to the neat PLA. $\Delta\sigma_{UTS}$ is the percentage difference in ultimate tensile strength compared to the neat PLA. $\Delta\sigma_y$ is the percentage difference in yield strength compared to the neat PLA. Δε is the percentage difference in elongation at break compared to the neat PLA.

Filament Type	MNPs (wt%)	E (Mpa)	ΔE(%)	σ_{UTS} (Mpa)	$\Delta\sigma_{UTS}$(%)	σy (Mpa)	Δσy (%)	ε at Break	Δε (%)
**EF.PLA**	0	1834		42		39		0.047	
**EF.PLA**	10	1186	-35	26	-38	23	-40	0.033	-30
**EF.PLA**	20	1130	-38	26	-38	23	-41	0.028	-41
**T. PLA**	0	1392		31		27		0.058	
**T. PLA**	10	1144	-18	28	-10	24	-12	0.039	-34
**T. PLA**	20	1091	-22	25	-20	21	-22	0.038	-35
**Pr. PLA**	0	1800		42		38		0.039	
**Pr. PLA**	10	1654	-8	33	-21	27	-29	0.035	-9
**Pr. PLA**	20	1567	-13	30	-28	24	-36	0.027	-30

To evaluate the mechanical performance of the filaments, stress-strain experiments were conducted, and the results are summarized in Table S1. The table presents the elastic modulus (E), ultimate tensile strength (σ_{UTS}), yield strength (σ_y), and elongation at break (ε) for three types of neat PLA materials (EasyFill PLA, Tough PLA, and Premium PLA). Additionally, these mechanical properties are reported for PLA filaments mixed with 10 and 20 wt% magnetic nanoparticles. For a comprehensive comparison, the percentage differences in these properties between the mixed PLA and the corresponding neat PLA are also provided.

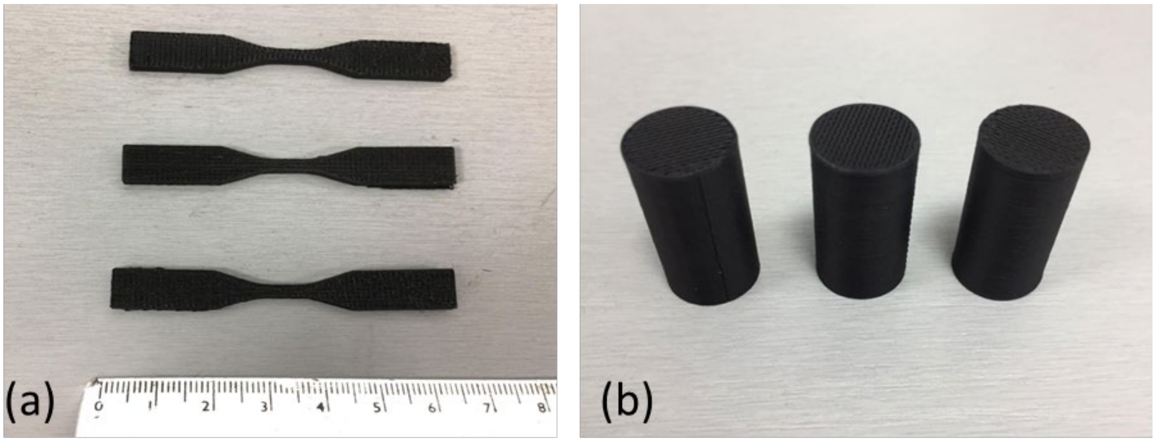

Figure S2: (a) Photograph of 3D-printed magnetic dog bone specimens with a ruler for scale, providing an estimation of their size. (b) Photograph of 3D-printed magnetic scaffolds. For each filament used, three dog bones and three magnetic scaffolds were printed to ensure reproducibility and standard deviation in the final results.
